# Burden of colon and rectum cancer attributable to processed meat consumption in China, 1990–2021

**DOI:** 10.3389/fnut.2025.1488077

**Published:** 2025-03-28

**Authors:** Zhouwei Zhan, Xiamei Chen, Jinping Zheng, Jingjie Xu, Sijing Zhou, Zengqing Guo, Bijuan Chen

**Affiliations:** ^1^Department of Medical Oncology, Clinical Oncology School of Fujian Medical University, Fujian Cancer Hospital, Fuzhou, China; ^2^Department of Operation, Clinical Oncology School of Fujian Medical University, Fujian Cancer Hospital, Fuzhou, China; ^3^Department of Radiation Oncology, Clinical Oncology School of Fujian Medical University, Fujian Cancer Hospital, Fuzhou, China

**Keywords:** colon and rectum cancer, processed meat, China, mortality, DALYs, epidemiology, Global Burden of Disease

## Abstract

**Background:**

Colon and rectum cancer (CRC) poses a significant public health challenge, and diets high in processed meat have been identified as key risk factors. This study aims to assess the burden of CRC attributable to processed meat consumption in China from 1990 to 2021, focusing on mortality, disability-adjusted life years (DALYs), years lived with disability (YLDs), and years of life lost (YLLs).

**Methods:**

Data were obtained from the Global Burden of Disease (GBD) study for CRC attributable to processed meat consumption in China from 1990 to 2021. The analysis included age-standardized rates for deaths, DALYs, YLDs, and YLLs, alongside age-period-cohort (APC) and decomposition analyses to examine temporal trends and contributing factors. Joinpoint regression was used to detect significant changes in trends.

**Results:**

The burden of CRC attributable to processed meat consumption in China increased significantly between 1990 and 2021. In 2021, males exhibited higher mortality and DALY rates than females, particularly in older age groups. The age-standardized death rate rose from 0.23 to 0.32 per 100,000, and DALYs increased from 5.83 to 8.57 per 100,000. Joinpoint analysis revealed steep rises in DALYs and YLLs, especially during 2007–2011. APC analysis showed that older cohorts consistently exhibited higher death rates, while more recent cohorts displayed lower mortality. Decomposition analysis indicated that population growth and epidemiological changes drove the rise in mortality, with aging contributing to a reduction in deaths. These factors had a more pronounced impact on males.

**Conclusion:**

The study highlights the growing burden of CRC linked to processed meat consumption in China over three decades, with significant gender differences and strong cohort effects. The findings call for targeted interventions to reduce processed meat consumption and mitigate CRC risk.

## Introduction

Colon and rectum cancer (CRC) remains a leading cause of cancer morbidity and mortality worldwide, with processed meat consumption recognized as a significant dietary risk factor (1, 2). Processed meat, which includes products such as bacon, sausages, and deli meats, undergoes preservation techniques like smoking, curing, or adding preservatives, leading to the formation of carcinogenic compounds such as nitrosamines (3, 4). Epidemiological studies have consistently demonstrated a strong association between processed meat intake and an increased risk of CRC (5), with the International Agency for Research on Cancer (IARC) classifying processed meat as a Group 1 carcinogen (1, 6). In China, changing dietary patterns over recent decades have resulted in a sharp increase in processed meat consumption, aligning with a concurrent rise in CRC incidence and mortality (7, 8).

The relationship between diet and CRC has prompted extensive research on dietary interventions aimed at reducing the burden of CRC (9–11). Although several studies have investigated the overall global impact of processed meat on CRC, less attention has been paid to the specific trends and burden within China, a country undergoing rapid dietary and epidemiological transitions (12). Despite global efforts to reduce CRC incidence through dietary guidelines and public health campaigns, the burden of CRC attributable to processed meat remains disproportionately high in China compared to other regions (13). These disparities are driven by differences in dietary habits, access to healthcare, and public awareness regarding the risks of processed meat consumption.

This study aims to assess the temporal trends and burden of CRC attributable to processed meat consumption in China from 1990 to 2021, using data from the Global Burden of Disease (GBD) study. By examining age-specific and sex-specific trends, and using decomposition and age-period-cohort (APC) analyses, this research seeks to provide a comprehensive understanding of how processed meat consumption has contributed to the rising burden of CRC in China. The findings will inform targeted public health strategies aimed at reducing CRC incidence through dietary modifications and policy interventions focused on processed meat consumption.

## Methods

### Data source

This study utilized data from the GBD Study 2021 to evaluate the burden of CRC attributable to a diet high in processed meat in China from 1990 to 2021. The GBD database integrates data from national dietary surveys, cohort studies, food availability data, and systematic reviews to estimate the incidence, prevalence, mortality, and disease burden, including disability-adjusted life years (DALYs), years lived with disability (YLDs), and years of life lost (YLLs) (14, 15). Data sources include government-led nutrition surveys such as the China Health and Nutrition Survey (CHNS), household food balance sheets from the Food and Agriculture Organization (FAO), and longitudinal cohort studies tracking dietary habits and CRC risk. The GBD study employs a standardized comparative risk assessment framework that integrates multiple data sources through Bayesian meta-regression (DisMod-MR 2.1). Each data source is adjusted for systematic differences by applying study-specific covariates that account for variations in study design, survey methodologies, and reporting biases. Data from cohort studies, dietary surveys, and food availability data are calibrated using statistical methods such as exposure modeling and cross-walking techniques. This approach ensures harmonization by aligning estimates from different methodologies to a common exposure scale. The spatiotemporal Gaussian process regression (ST-GPR) method is used to impute missing values, while Monte Carlo simulations generate uncertainty intervals (UIs) reflecting variability in source reliability. When conflicting data are encountered, preference is given to higher-quality data sources based on predefined selection criteria, and sensitivity analyses are conducted to evaluate the robustness of estimates (14, 15). As this study utilized publicly available secondary data, ethical approval and informed consent were not required.

### Definition and estimation of processed meat intake

Processed meat is defined as any meat product preserved through smoking, curing, salting, or the addition of chemical preservatives, such as bacon, sausages, ham, and deli meats. The GBD study estimates population-level exposure to processed meat rather than individual dietary intake, using a Comparative Risk Assessment (CRA) framework (14). Dietary exposure data are aggregated from national dietary surveys, food availability datasets, and published literature to estimate intake levels. A dose–response meta-analysis of epidemiological studies quantifies the relative risk (RR) of CRC associated with processed meat consumption. The population attributable fraction (PAF) is then calculated using the formula: $PAF=\frac{P\times \left(RR-1\right)}{P\times \left(RR-1\right)+1}$, where P represents the proportion of the population exposed. Finally, Monte Carlo simulations generate 95% UIs for all estimates, ensuring robust quantification of uncertainty (14).

The CRA framework does not explicitly account for individual-level confounders such as smoking, obesity, and alcohol consumption in its exposure assessment, as its primary objective is to estimate population-level disease burden attributable to specific risk factors in a standardized manner. However, these confounders are implicitly adjusted for through the relative risks (RRs) derived from meta-analyses and large-scale cohort studies, which typically control for major confounders in their statistical models. While residual confounding cannot be entirely ruled out, the Monte Carlo simulation approach and UIs account for variability in data sources, ensuring that the estimated burden of CRC due to processed meat consumption reflects the most robust and methodologically consistent approach within the GBD framework. Additionally, processed meats contain carcinogenic compounds, including nitrates, nitrites, heterocyclic amines (HCAs), and polycyclic aromatic hydrocarbons (PAHs), which have been strongly linked to CRC development through multiple biological pathways (1, 13).

### Descriptive analysis

Descriptive analysis was conducted to provide a basic understanding of the temporal trends in CRC burden attributable to processed meat consumption. Summary statistics, including the total number of CRC cases, deaths, and burden metrics such as DALYs, YLDs, and YLLs, were calculated for different age groups and sex. These descriptive statistics established the foundation for subsequent trend analysis and helped identify initial patterns in the data.

### Joinpoint regression analysis

Joinpoint regression analysis was conducted to identify statistically significant changes in trends over time for the age-standardized rates of CRC deaths, DALYs, YLDs, and YLLs attributable to processed meat consumption. This method allows for the detection of points where significant shifts in trends occur, providing a more precise characterization of long-term patterns. To perform this analysis, we utilized the Joinpoint Regression Program (version 5.2.0), which employs a grid-search method to fit segmented regression models with unknown breakpoints. The algorithm starts with the simplest model (a single continuous trend) and iteratively tests whether additional joinpoints significantly improve the model fit. The selection of the optimal number of joinpoints is determined by Monte Carlo permutation tests, which compare models with varying numbers of joinpoints while controlling for multiple testing using a Bonferroni correction. The regression models were fitted using a log-linear approach, allowing for the estimation of annual percent change (APC) and average annual percent change (AAPC) in CRC burden. The models incorporated Poisson variance assumptions to account for heteroscedasticity in age-adjusted rates. APCs were calculated for each identified trend segment, with 95% confidence intervals (CIs) estimated to quantify the precision of trend estimates (16, 17). A statistically significant change in trend was defined as one where *p* < 0.05. The number and location of joinpoints were determined through an iterative grid-search method, ensuring that the optimal model captures meaningful inflection points in CRC burden trends.

### Age-period-cohort analysis

To assess the distinct impacts of age, time period, and birth cohort on CRC mortality trends related to processed meat consumption, an APC model was applied. This approach allows for an independent evaluation of each factor’s contribution to observed patterns. Given the inherent collinearity among age, period, and cohort variables, the intrinsic estimator (IE) method was used to provide more accurate effect estimates. Mortality data spanning 1990 to 2021 were retrieved from the GBD database, categorized into five-year age groups, along with annual population estimates. For analytical consistency, individuals younger than 5 and those older than 95 were grouped together. Age classifications followed a structured format (e.g., 0–4, 5–9, 10–14), with the youngest category designated as “0.” Cumulative incidence and mortality rates were computed across five-year intervals (e.g., 1992–1996, 1997–2001) (18, 19). The APC model was implemented using the Epi package (version 2.46) in R (version 4.3.1), with residual deviations and Akaike Information Criterion (AIC) values examined to determine the optimal model.

### Decomposition analysis

Decomposition analysis was conducted to quantify the contributions of aging, epidemiological changes, and population growth to the trends in CRC mortality attributable to processed meat consumption. The method decomposes changes in mortality into these three components, enabling a clearer understanding of the forces driving the changes over time (20). Aging refers to the increasing proportion of older individuals in the population, which naturally raises the risk of CRC due to the higher incidence of cancer in older age groups. Epidemiological changes encompass shifts in dietary patterns, lifestyle factors (such as smoking and alcohol use), and improvements in healthcare (such as early detection and cancer treatment), which can either increase or decrease CRC mortality. Population growth reflects the overall increase in population size, which increases the number of individuals at risk for CRC, thus influencing mortality trends. The analysis was performed for both sexes combined and separately for males and females, highlighting sex-specific patterns in mortality changes.

## Results

### Burden of CRC attributable to a diet high in processed meat in China, 2021

**Figure 1:**
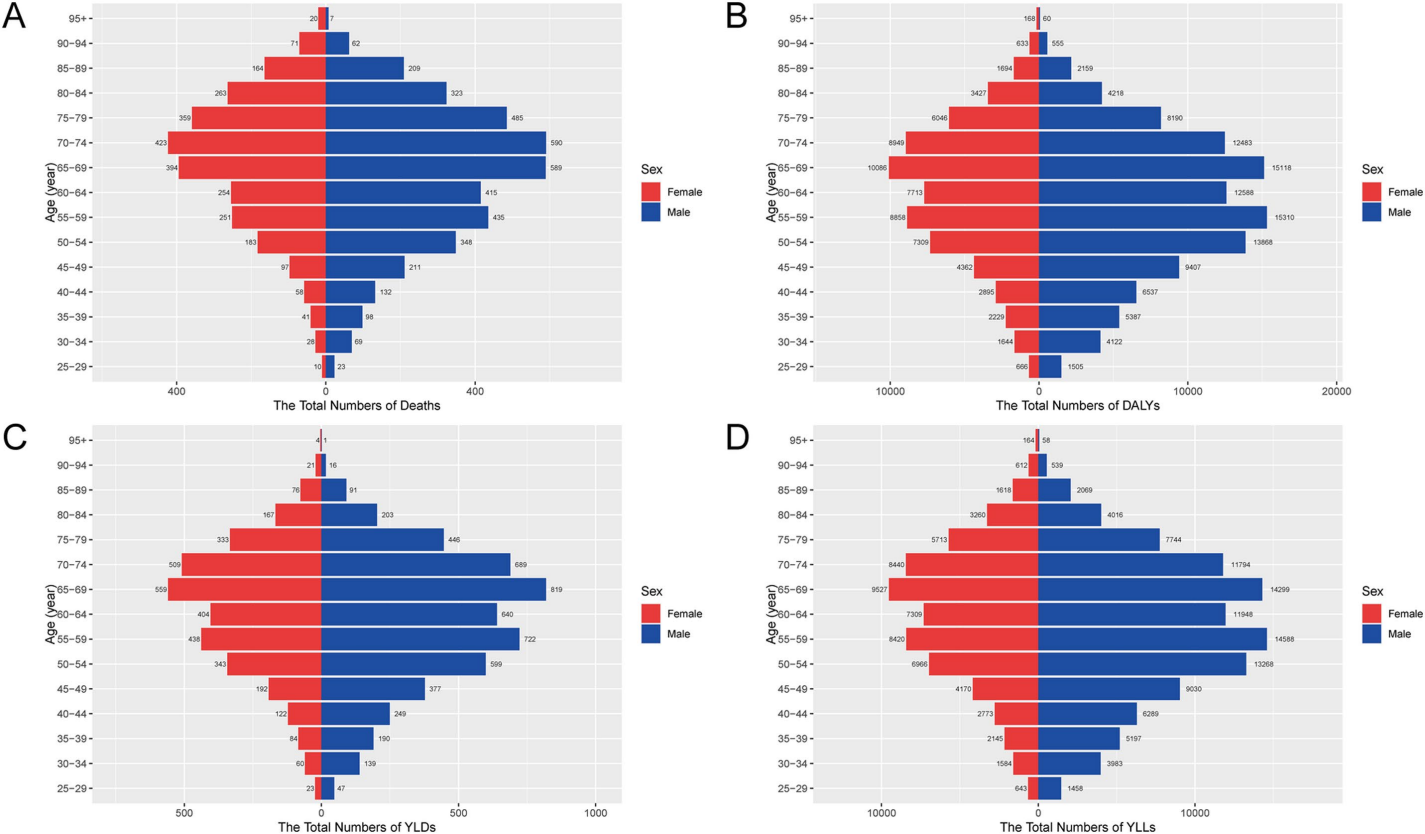
Age- and sex-specific numbers of deaths, DALYs, YLDs, and YLLs for CRC attributable to a diet high in processed meat in China, 2021. **(A)** Numbers of deaths by age group and sex. **(B)** Numbers of DALYs. **(C)** Numbers of YLDs. **(D)** Numbers of YLLs. Abbreviations: DALYs, disability-adjusted life years; YLDs, years lived with disability; YLLs, years of life lost.

**Figure 2:**
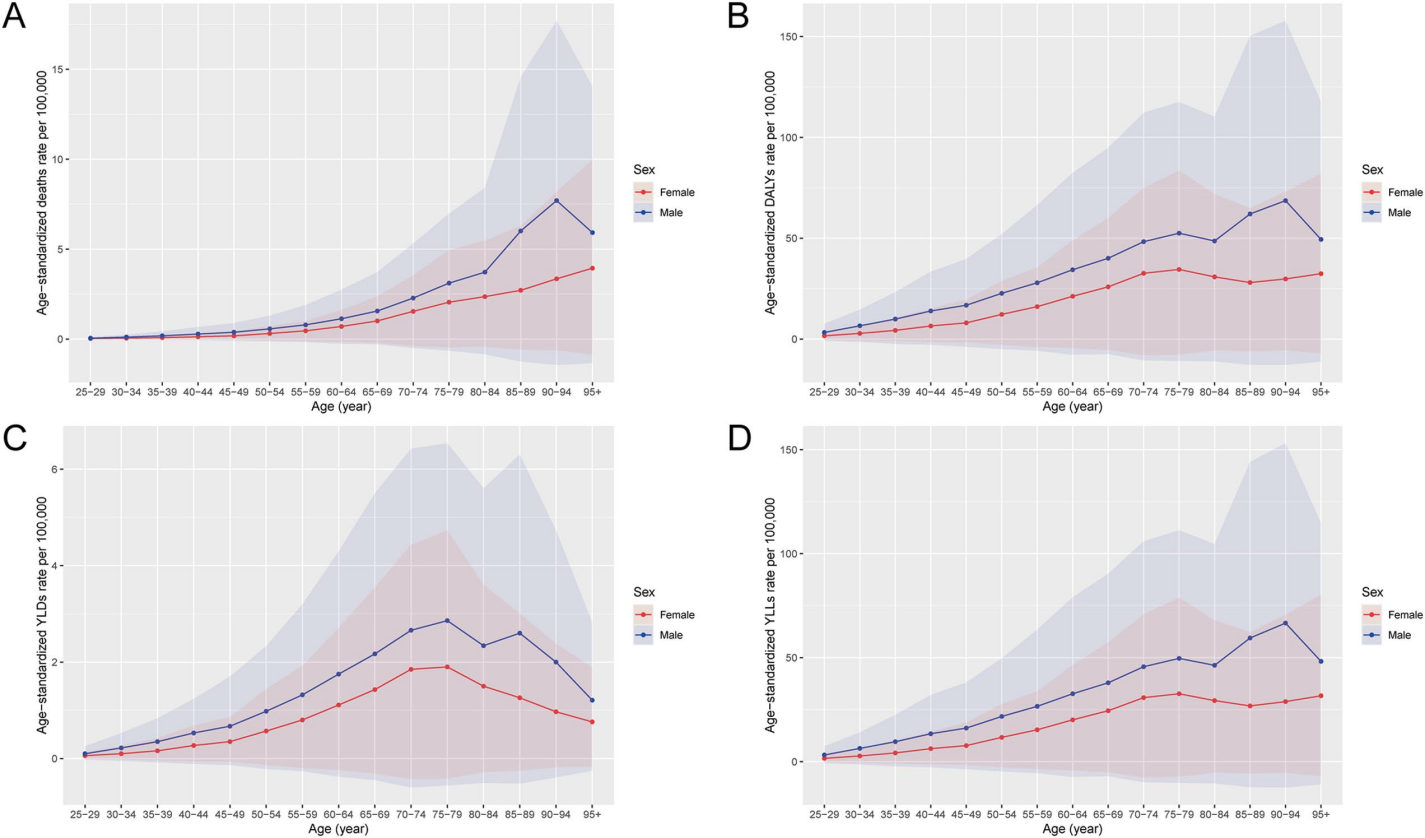
Age- and sex-specific rates of deaths, DALYs, YLDs, and YLLs for CRC attributable to a diet high in processed meat in China, 2021. **(A)** The rate of deaths by age group and sex. **(B)** The rate of DALYs. **(C)** The rate of YLDs. **(D)** The rate of YLLs. Rates are expressed per 100,000 population. The solid lines represent the estimated trends, and the shaded areas indicate 95% uncertainty intervals (UIs), estimated using Monte Carlo simulations. Abbreviations: DALYs, disability-adjusted life years; YLDs, years lived with disability; YLLs, years of life lost.

In 2021, the burden of CRC attributable to a diet high in processed meat in China was characterized by significant gender differences, as shown in both Figure 1 and Figure 2. The total number of deaths due to CRC was higher among males than females across all age groups, with males exhibiting a substantially larger number of deaths in older age groups (Figure 1A). Correspondingly, the rate of deaths also followed this trend, with males experiencing a markedly higher mortality rate compared to females, particularly among those aged 60 and above (Figure 2A). The overall impact of CRC mortality was reflected in the age-standardized death rates from Table 1, where the male population had a death rate of 0.42 per 100,000 people, which was higher than that of females at 0.24 per 100,000 people.

**Table 1:** All-age cases and age-standardized deaths, DALYs, YLDs, and YLLs rates in 2021 for CRC attributable to diet high in processed meat in China.

Measure	All-ages cases			Age-standardized rates per 100,000 people		
	Total	Male	Female	Total	Male	Female
Deaths	6,612 (−1,355, 14,767)	3,997 (−848, 9,226)	2,615 (−571, 5,848)	0.32 (−0.07, 0.71)	0.42 (−0.09, 0.94)	0.24 (−0.05, 0.54)
DALYs	178,187 (−37,003, 405,025)	111,507 (−23,736, 258,266)	66,679 (−15,019, 147,750)	8.57 (−1.78, 19.35)	11.03 (−2.35, 25.22)	6.22 (−1.4, 13.76)
YLDs	8,561 (−1847, 19,709)	5,228 (−1,096, 12,145)	3,333 (−766, 7,632)	0.4 (−0.09, 0.93)	0.51 (−0.11, 1.17)	0.3 (−0.07, 0.71)
YLLs	169,626 (−35,117, 387,552)	106,279 (−22,563, 245,272)	63,347 (−14,191, 140,194)	8.16 (−1.69, 18.45)	10.52 (−2.24, 23.98)	5.91 (−1.32, 13.04)

Values in parentheses indicate 95% uncertainty intervals (UIs), estimated using Monte Carlo simulations. Data are sourced from the GBD database, where precision and rounding vary based on post-statistical processing. CRC, colon and rectum cancer; DALYs, disability-adjusted life-years; YLDs, years lived with disability; YLLs, years of life lost.

The total DALYs also revealed a higher burden on males, with a more pronounced increase in middle-aged and older populations (Figure 1B). The rate of DALYs was consistently higher among males, showing a significant difference, especially in the 60–79 age range (Figure 2B). The age-standardized DALY rates followed this pattern, with males having an average of 11.03 per 100,000 people, compared to 6.22 per 100,000 for females, reflecting the higher burden of CRC attributable to processed meat among men (Table 1). The non-fatal burden of CRC, measured by YLDs, showed a similar trend but with smaller gender differences (Figure 1C). Males still experienced a slightly higher burden, with more YLDs concentrated in older populations (Figure 2C). In terms of premature mortality, measured by YLLs, males experienced significantly higher rates across all age groups, with a sharp increase starting from age 55 (Figure 1D, Figure 2D). This gender disparity was also reflected in the age-standardized YLL rates, where males had a rate of 10.52 per 100,000 people compared to 5.91 per 100,000 for females (Table 1).

### Temporal trends in CRC burden attributable to a diet high in processed meat, 1990–2021

**Figure 3:**
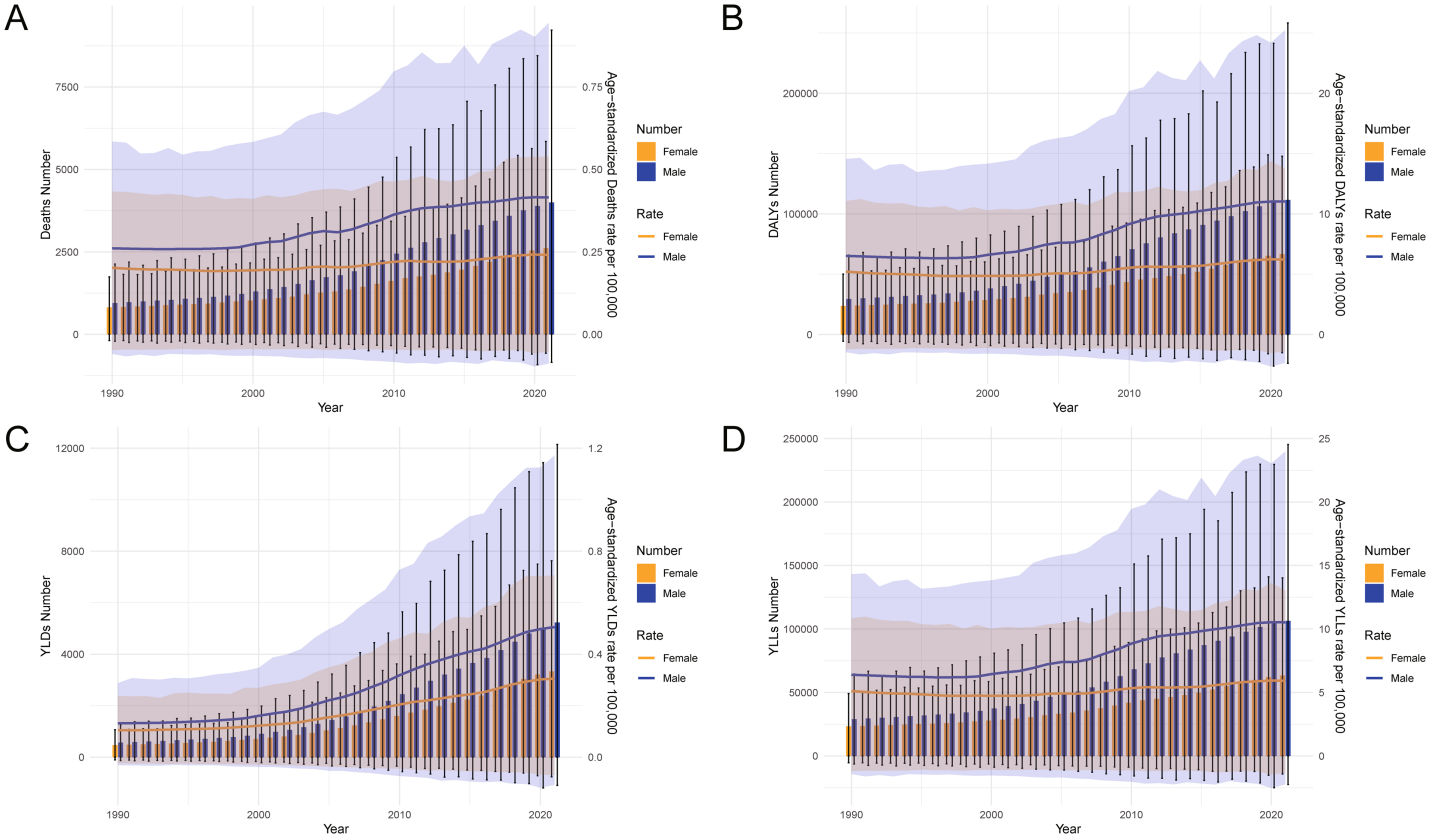
Temporal trends in the numbers and age-standardized rates of deaths, DALYs, YLDs, and YLLs for CRC attributable to a diet high in processed meat in China, 1990–2021. **(A)** Number of deaths and ASMRs over time. **(B)** Number of DALYs and age-standardized DALY rates. **(C)** Number of YLDs and age-standardized YLD rates. **(D)** Number of YLLs and age-standardized YLL rates. Error bars and shaded areas represent 95% uncertainty intervals (UIs), estimated using Monte Carlo simulations. Abbreviations: DALYs, disability-adjusted life years; YLDs, years lived with disability; YLLs, years of life lost; ASMR, age-standardized mortality rate.

From 1990 to 2021, the burden of CRC attributable to a diet high in processed meat in China exhibited a consistent upward trend in both the numbers and age-standardized rates of deaths, DALYs, YLDs, and YLLs for both males and females (Figure 3). The number of deaths and age-standardized death rates increased steadily over time, with males consistently experiencing higher rates than females (Figure 3A). This trend was also reflected in the DALYs, where both the number and rate of DALYs showed a sharp rise, particularly in males (Figure 3B). The non-fatal burden, represented by YLDs, demonstrated a similar increasing pattern, with males showing higher numbers and rates across the period (Figure 3C). The premature mortality impact of CRC, as measured by YLLs, also escalated significantly, with a marked increase in both the number and rate of YLLs, particularly in males, who consistently exhibited higher values compared to females (Figure 3D). These trends highlight the growing public health burden of CRC attributable to processed meat consumption over the past three decades, with a more substantial impact on males.

### Age-specific changes in CRC burden attributable to a diet high in processed meat, 1990 and 2021

**Figure 4:**
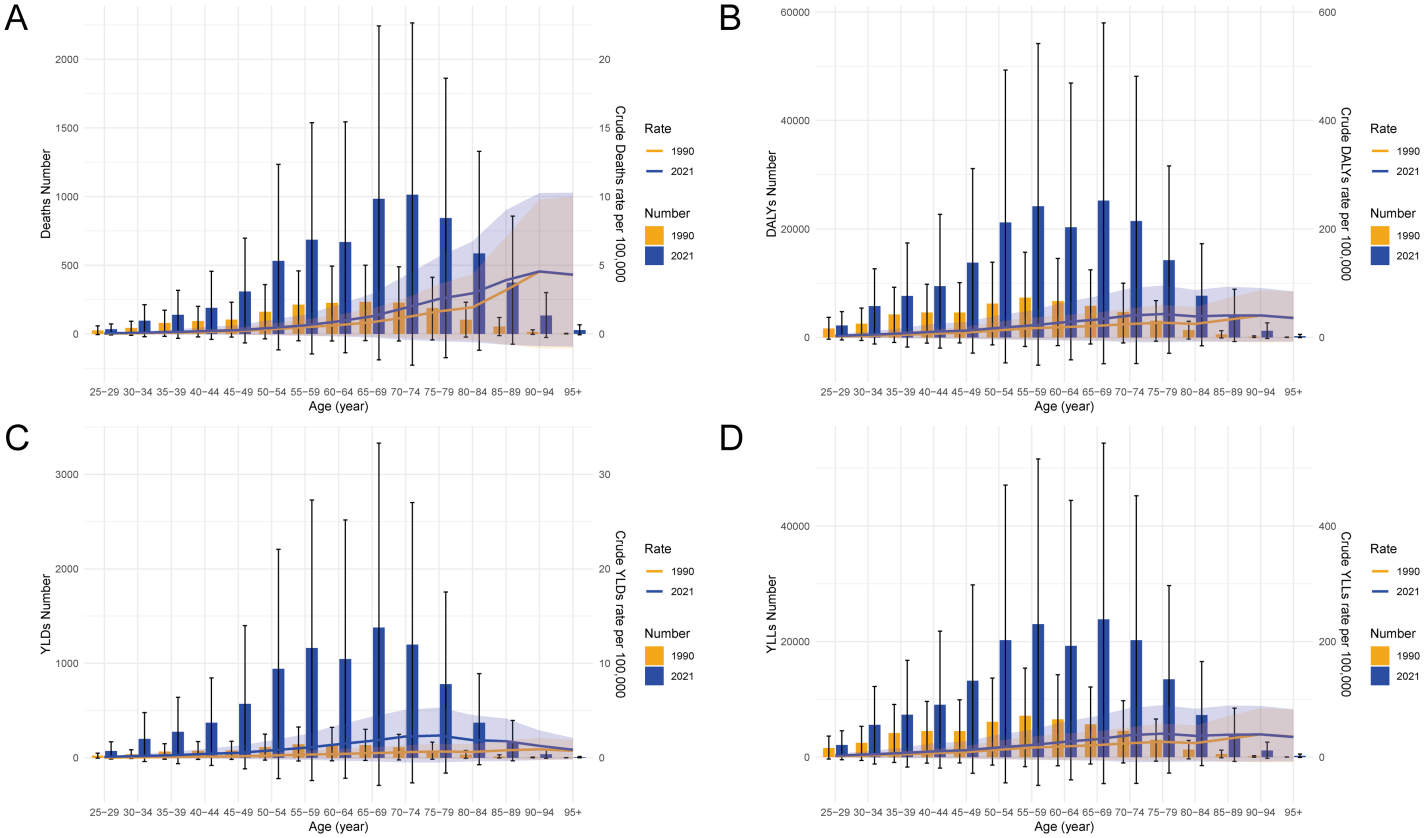
Age-specific comparisons of numbers and crude rates of deaths, DALYs, YLDs, and YLLs for CRC attributable to a diet high in processed meat in China in 1990 and 2021. **(A)** Crude rates and numbers of deaths. **(B)** Crude rates and numbers of DALYs. **(C)** Crude rates and numbers of YLDs. **(D)** Crude rates and numbers of YLLs. Error bars and shaded areas represent 95% uncertainty intervals (UIs), estimated using Monte Carlo simulations. Abbreviations: DALYs, disability-adjusted life years; YLDs, years lived with disability; YLLs, years of life lost.

Between 1990 and 2021, the burden of CRC attributable to a diet high in processed meat in China increased substantially across all age groups, as demonstrated by the data in Figure 4. The number of deaths and the crude death rates rose significantly in 2021 compared to 1990, with older age groups exhibiting the highest increases (Figure 4A). This trend was mirrored in the DALYs, where both the numbers and crude rates showed a marked rise, particularly among middle-aged and older populations, reflecting the growing overall burden of CRC (Figure 4B). The non-fatal burden, represented by YLDs, also increased in 2021 across all age groups, with a noticeable concentration of YLDs in older populations (Figure 4C). Similarly, YLLs, which measure premature mortality, saw a sharp escalation in both the numbers and crude rates from 1990 to 2021, with the greatest burden observed in the elderly population (Figure 4D). These data underscore the escalating impact of CRC related to processed meat consumption over the past three decades, with older age groups bearing the brunt of the burden.

### Changes in CRC burden attributable to a diet high in processed meat: China vs. global, 1990–2021

**Figure 5:**
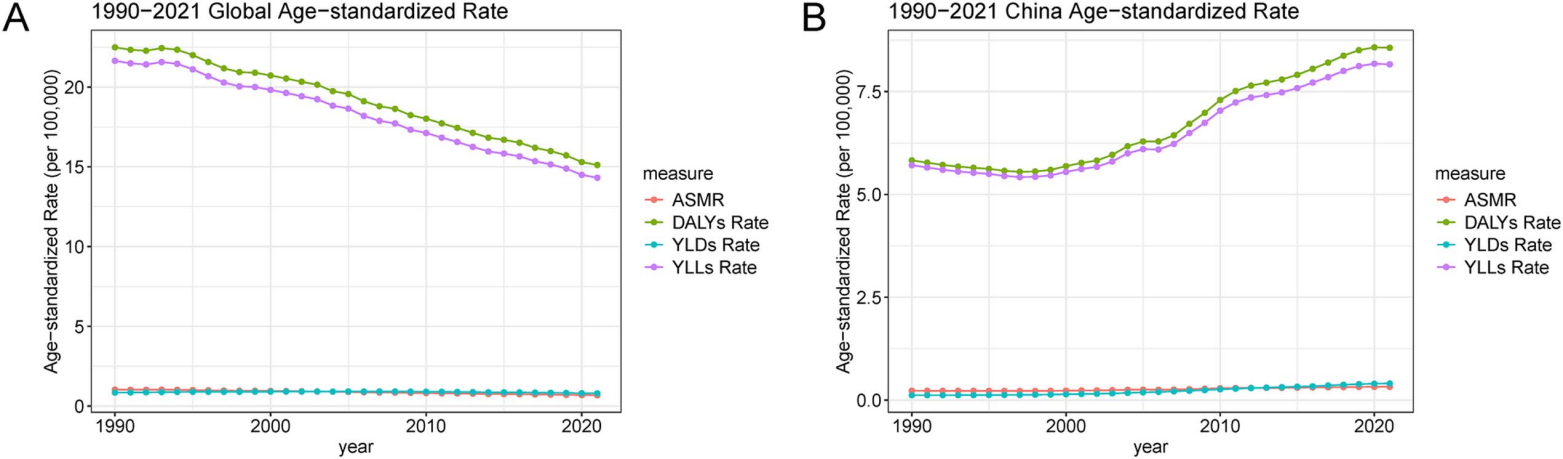
Global and China age-standardized rates for deaths, DALYs, YLDs, and YLLs due to CRC attributable to a diet high in processed meat from 1990 to 2021. **(A)** Global trends in ASMR, DALYs, YLDs, and YLLs. **(B)** China-specific trends in age-standardized rates for ASMR, DALYs, YLDs, and YLLs. Abbreviations: DALYs, disability-adjusted life years; YLDs, years lived with disability; YLLs, years of life lost; ASMR, age-standardized mortality rate.

Between 1990 and 2021, the age-standardized rates for CRC attributable to a diet high in processed meat showed distinct trends in China compared to global patterns, as illustrated in Table 2 and Figure 5. In China, there was a significant increase in the age-standardized death rate, which rose from 0.23 per 100,000 in 1990 to 0.32 per 100,000 in 2021, reflecting a growing mortality burden (Table 2). Conversely, globally, the age-standardized death rate decreased, indicating a divergence in trends. Similarly, the age-standardized DALYs rate in China increased significantly from 5.83 to 8.57 per 100,000, while globally, it decreased, highlighting a rising burden in China. This pattern was consistent across YLDs and YLLs, with China showing a marked increase, particularly in YLDs, which grew from 0.12 to 0.40 per 100,000, compared to a slight global decline. Figure 5 further underscores these trends, showing a continuous increase in age-standardized rates for deaths, DALYs, YLDs, and YLLs in China, contrasting with global rates that generally declined over the same period. These findings indicate that China is experiencing a growing public health challenge related to CRC and processed meat consumption, whereas the global burden shows signs of improvement.

**Table 2:** Change of age-standardized rates in deaths, DALYs, YLDs, and YLLs for CRC attributable to diet high in processed meat between 1990 and 2021 in China and Global level.

Measure	China			Global		
	1990	2021	Change	1990	2021	Change
Deaths	0.23 (−0.05, 0.48)	0.32 (−0.07, 0.71)	1.17 (1.00–1.34)^*^	1.03 (−0.25, 2.09)	0.68 (−0.16, 1.4)	−1.31 (−1.44 - −1.19)^*^
DALYs	5.83 (−1.37, 12.56)	8.57 (−1.78, 19.35)	1.30 (1.15–1.45)^*^	22.49 (−5.52, 45.73)	15.11 (−3.6, 30.93)	−1.25 (−1.38 - −1.12)^*^
YLDs	0.12 (−0.03, 0.27)	0.4 (−0.09, 0.93)	4.11 (3.92–4.29)^*^	0.85 (−0.22, 1.81)	0.8 (−0.2, 1.74)	−0.19 (−0.26 - −0.13)^*^
YLLs	5.71 (−1.34, 12.32)	8.16 (−1.69, 18.45)	1.21 (1.06–1.35)^*^	21.64 (−5.31, 44.01)	14.32 (−3.39, 29.44)	−1.30 (−1.44 - −1.17)^*^

Values in parentheses indicate 95% uncertainty intervals (UIs), estimated using Monte Carlo simulations. Negative lower bounds in UIs reflect statistical uncertainty and should not be interpreted as actual negative values. Data are sourced from the GBD database, where precision and rounding vary based on post-statistical processing. CRC, colon and rectum cancer; DALYs, disability-adjusted life-years; YLDs, years lived with disability; YLLs, years of life lost; ^*^p < 0.05.

### Temporal trends in age-standardized CRC burden based on joinpoint analysis, 1990–2021

**Figure 6:**
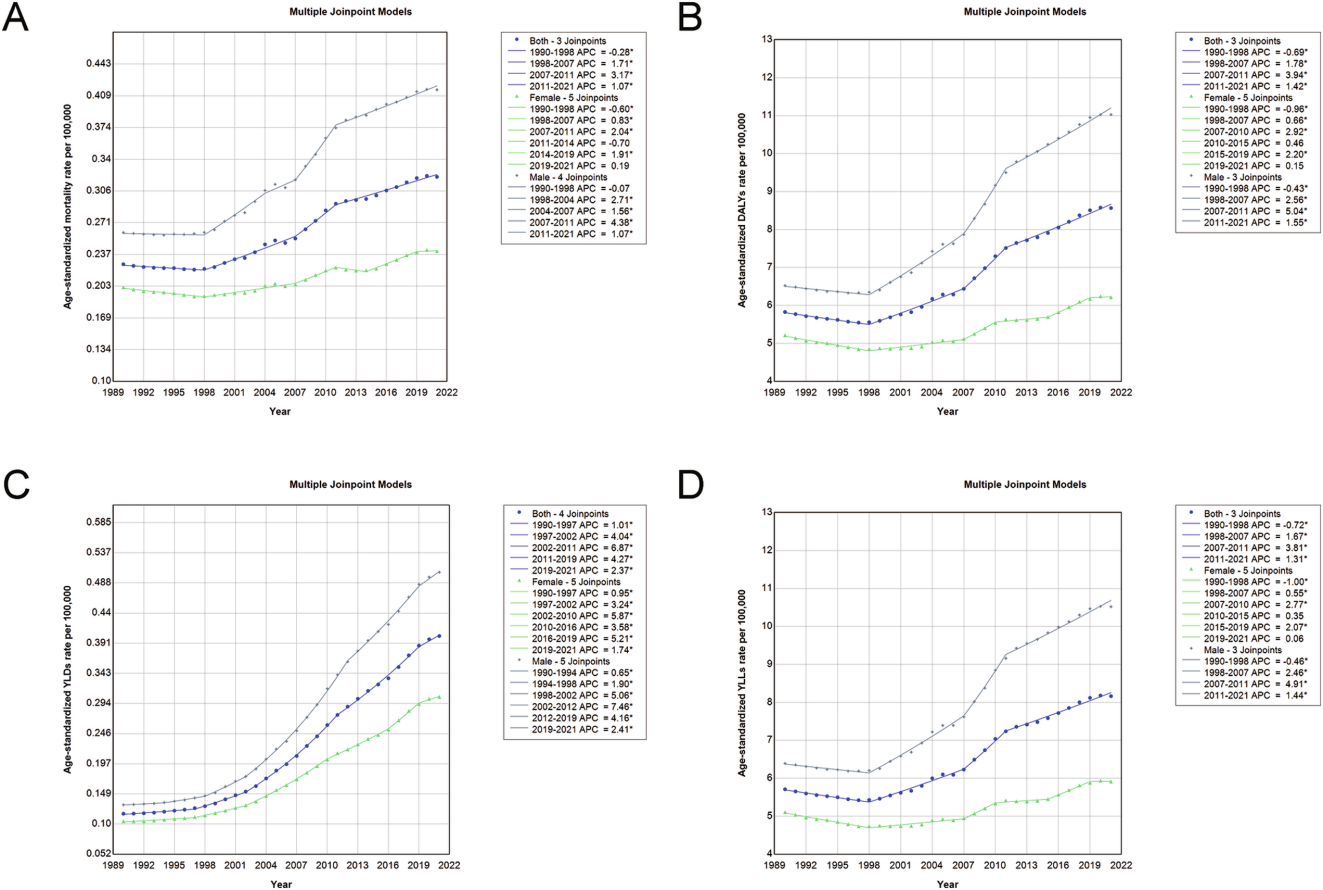
Joinpoint analysis of age-standardized rates for deaths, DALYs, YLDs, and YLLs due to CRC attributable to a diet high in processed meat in China, 1990–2021. **(A)** Joinpoint analysis of ASMRs. **(B)** Joinpoint analysis of age-standardized DALY rates. **(C)** Joinpoint analysis of age-standardized YLD rates. **(D)** Joinpoint analysis of age-standardized YLL rates. Asterisk (*) denotes statistically significant trend changes (*p* < 0.05) as identified by Joinpoint regression analysis. Abbreviations: DALYs, disability-adjusted life years; YLDs, years lived with disability; YLLs, years of life lost; ASMR, age-standardized mortality rate.

The joinpoint analysis of age-standardized rates for CRC attributable to a diet high in processed meat in China from 1990 to 2021 reveals several key trends (Figure 6 and Table 3). For both sexes, the age-standardized mortality rate (ASMR) showed significant changes over time, with an initial slight decline from 1990 to 1998, followed by an increasing trend, particularly after 2007. For DALYs and YLLs, the burden escalated substantially during 2007–2011, with the rate of increase stabilizing somewhat in the following decade. YLDs, which measure the non-fatal burden of CRC, saw consistent growth throughout the period, with notable spikes in the 2002–2011 range. The trends differed between genders, with males experiencing steeper increases in ASMR, DALYs, and YLLs during certain periods. From 1998 to 2007, males saw a significant acceleration in these rates, followed by even sharper increases between 2007 and 2011. In contrast, females displayed a more moderate growth in burden, though they also experienced a notable rise in DALYs and YLLs during the same periods. These patterns reflect the growing burden of CRC attributable to processed meat consumption, particularly in men, driven largely by both mortality and premature loss of life (Table 3).

**Table 3:** Trends in age-standardized mortality, DALY, YLD, and YLL rates (per 100,000 persons) among both sexes, males, and females from 1990 to 2021 for CRC attributable to diet high in processed meat in China.

	Age-standardized mortality rate			Age-standardized DALY rate			Age-standardized YLD rate			Age-standardized YLL rate		
Gender	Period	APC (95% CI)	AAPC (95% CI)	Period	APC (95% CI)	AAPC (95% CI)	Period	APC (95% CI)	AAPC (95% CI)	Period	APC (95% CI)	AAPC (95% CI)
Both	1990–1998	−0.28 (−0.52 - −0.05)^*^	1.17 (1.00–1.34)^*^	1990–1998	−0.69 (−0.90 - –0.48)^*^	1.30 (1.15–1.45)^*^	1990–1997	1.01 (0.76–1.27)^*^	4.11 (3.92–4.29)^*^	1990–1998	−0.72 (−0.93 - −0.51)	1.21 (1.06–1.35)^*^
	1998–2007	1.71 (1.47–1.96)^*^		1998–2007	1.78 (1.57–2.00)^*^		1997–2002	4.04 (3.41–4.67)^*^		1998–2007	1.68 (1.46–1.89)	
	2007–2011	3.17 (2.05–4.30)^*^		2007–2011	3.94 (2.95–4.95)^*^		2002–2011	6.87 (6.65–7.09)^*^		2007–2011	3.81 (2.83–4.80)	
	2011–2021	1.07 (0.90–1.24)^*^		2011–2021	1.42 (1.27–1.58)^*^		2011–2019	4.27 (3.99–4.54)^*^		2011–2021	1.31 (1.16–1.46)	
							2019–2021	2.37 (0.40–4.39)^*^				
Female	1990–1998	−0.60 (−0.82 - −0.39)^*^	0.59 (0.32–0.87)^*^	1990–1998	−0.96 (−1.16 - –0.77)^*^	0.59 (0.34–0.84)^*^	1990–1997	0.95 (0.75–1.14)	3.54 (3.35–3.74)^*^	1990–1998	−1.00 (−1.19 - −0.81)^*^	0.49 (0.24–0.74)^*^
	1998–2007	0.83 (0.61–1.05)^*^		1998–2007	0.66 (0.47–0.86)^*^		1997–2002	3.24 (2.79–3.70)		1998–2007	0.55 (0.35–0.74) ^*^	
	2007–2011	2.04 (1.06–3.03)^*^		2007–2010	2.92 (1.08–4.79)^*^		2002–2010	5.87 (5.67–6.08)		2007–2010	2.77 (0.98–4.60) ^*^	
	2011–2014	−0.70 (−2.63–1.27)		2010–2015	0.46 (−0.10–1.03)		2010–2016	3.58 (3.25–3.91)		2010–2015	0.35 (−0.20–0.90)	
	2014–2019	1.91 (1.27–2.55)^*^		2015–2019	2.20 (1.26–3.14)^*^		2016–2019	5.21 (3.70–6.74)		2015–2019	2.07 (1.15–3.00) ^*^	
	2019–2021	0.19 (−1.83–2.25)		2019–2021	0.15 (−1.73–2.06)		2019–2021	1.74 (0.26–3.24)		2019–2021	0.06 (−1.78–1.93)	
Male	1990–1998	−0.07 (−0.24–0.09)	1.56 (1.37–1.74)^*^	1990–1998	−0.43 (−0.65 - –0.20)^*^	1.77 (1.61–1.93)^*^	1990–1994	0.65 (0.00–1.30)^*^	4.46 (4.20–4.71) ^*^	1990–1998	−0.46 (−0.68 - −0.23) ^*^	1.68 (1.52–1.84)^*^
	1998–2004	2.71 (2.36–3.07)^*^		1998–2007	2.56 (2.33–2.79)^*^		1994–1998	1.90 (0.87–2.94)^*^		1998–2007	2.46 (2.23–2.69)^*^	
	2004–2007	1.56 (0.03–3.11)^*^		2007–2011	5.04 (3.96–6.13)^*^		1998–2002	5.06 (3.99–6.14)^*^		2007–2011	4.91 (3.83–6.00)^*^	
	2007–2011	4.38 (3.58–5.17)^*^		2011–2021	1.55 (1.38–1.71)^*^		2002–2012	7.46 (7.26–7.66)^*^		2011–2021	1.44 (1.28–1.61)^*^	
	2011–2021	1.07 (0.95–1.19)^*^					2012–2019	4.16 (3.80–4.53)^*^				
							2019–2021	2.41 (0.32–4.55)^*^				

CRC, colon and rectum cancer; AAPC, average annual percent change presented for full period; APC, annual percent change; CI, confidence interval. ^*^p < 0.05.

### Age-period-cohort analysis of CRC death rates attributable to processed meat consumption

**Figure 7:**
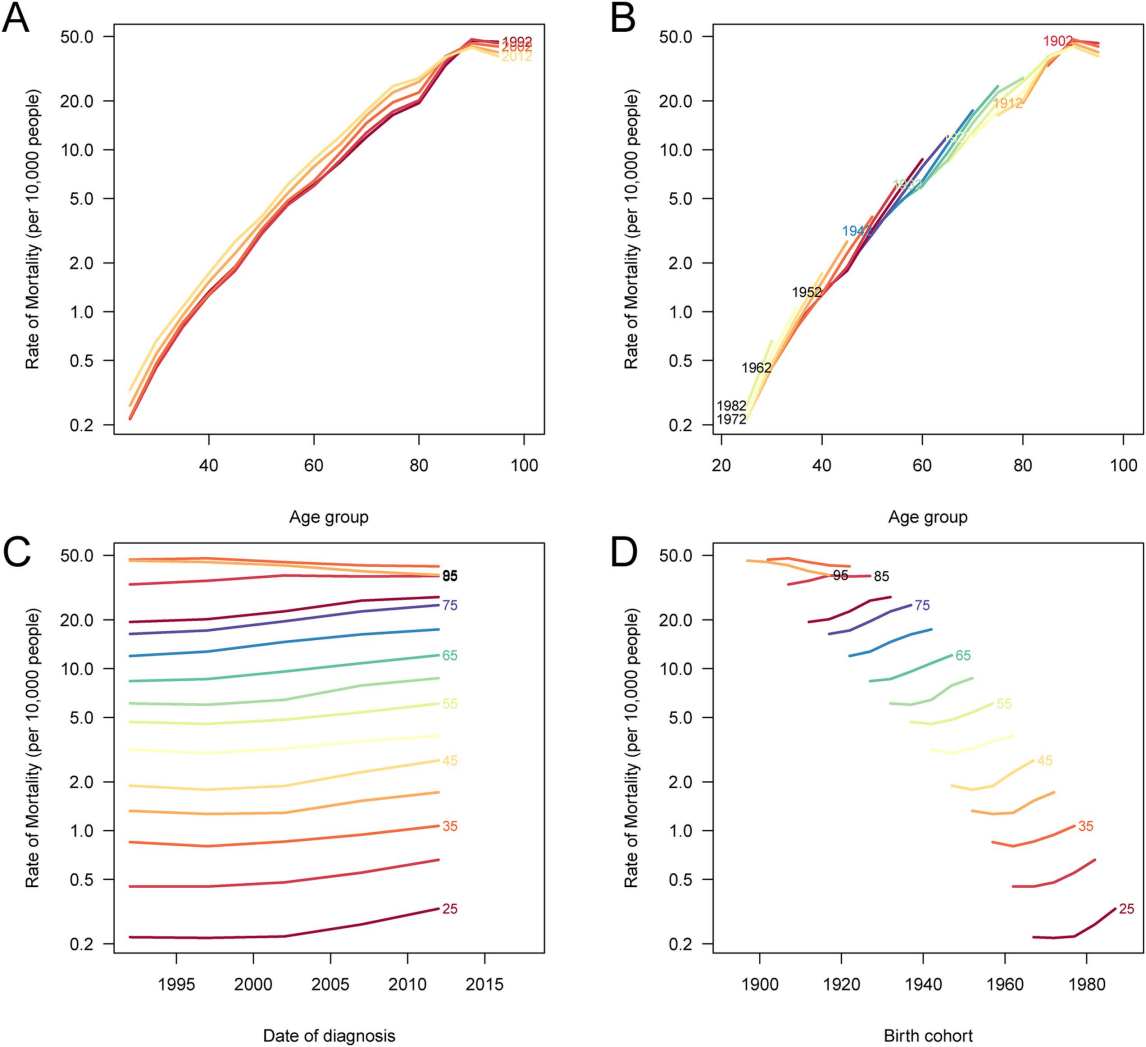
Age-period-cohort analysis of age-specific death rates for CRC attributable to a diet high in processed meat in China. **(A)** Age-specific death rates according to time periods, where each line connects the age-specific death rates for a 5-year period. **(B)** Age-specific death rates according to birth cohorts, with each line connecting the age-specific death rates for a 5-year birth cohort. **(C)** Period-specific death rates according to age groups, where each line connects the period-specific death rates for a 5-year age group. **(D)** Birth cohort-specific death rates according to age groups, where each line connects the cohort-specific death rates for a 5-year age group.

The age-period-cohort (APC) analysis for CRC deaths attributable to processed meat consumption in China reveals distinct patterns based on age groups, time periods, and birth cohorts (Figure 7). The age-specific death rates indicate that older age groups consistently exhibit higher mortality rates across all time periods, with a noticeable increase in mortality beginning from middle age onwards (Figure 7A). When analyzed by birth cohort, the results show that more recent cohorts tend to have lower CRC death rates, particularly in younger age groups, compared to older cohorts who experience higher mortality as they age (Figure 7B). The period-specific death rates demonstrate temporal variations, with more recent time periods showing an upward shift in CRC mortality rates across different age groups (Figure 7C). Finally, the cohort-specific death rates, when viewed across various age groups, highlight that older cohorts have consistently higher death rates as they progress through life, particularly in later age groups (Figure 7D). These trends illustrate the influence of age, birth cohort, and time period on CRC mortality in China.

### Decomposition analysis of CRC deaths attributable to processed meat consumption

**Figure 8:**
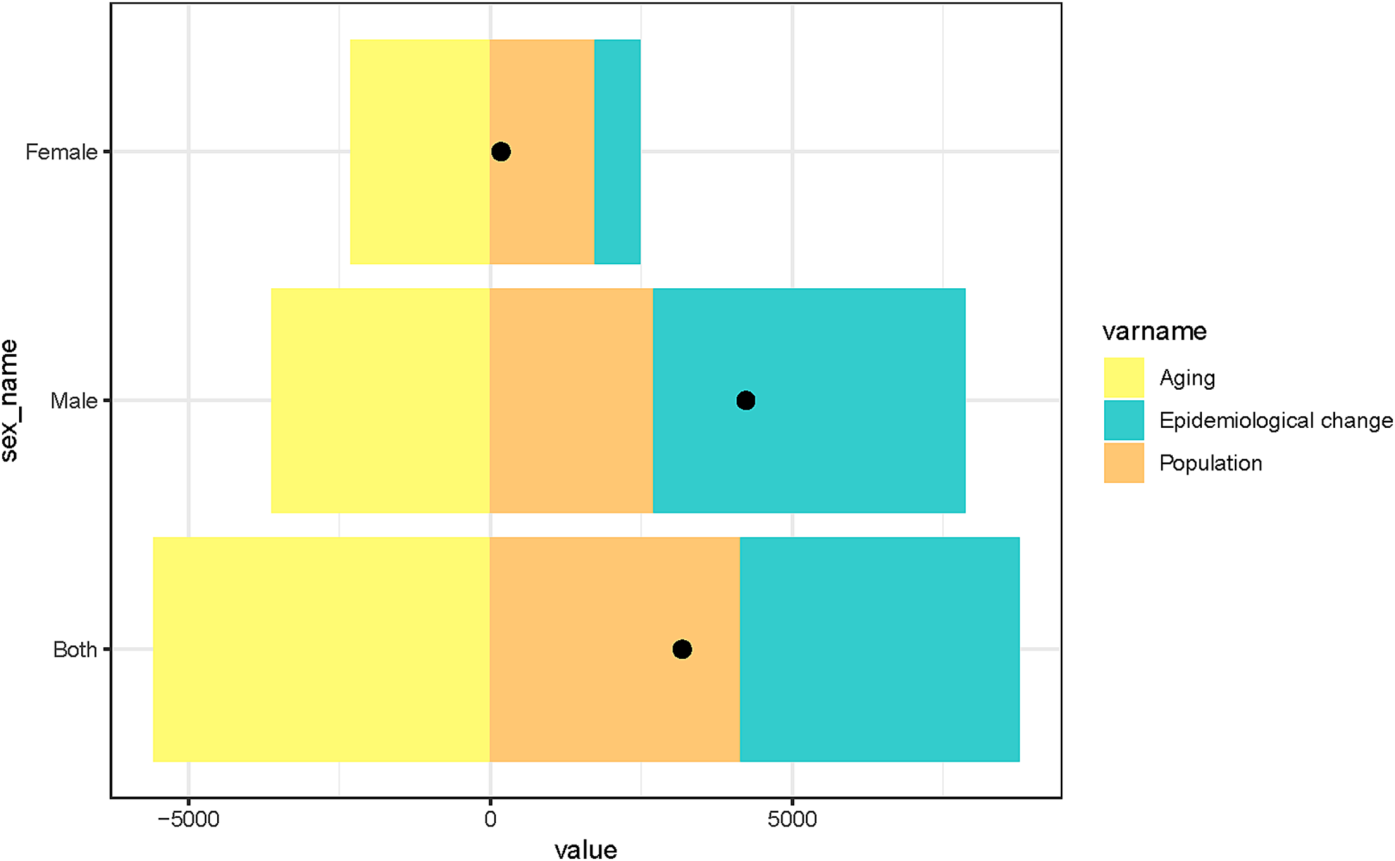
Decomposition analysis of factors contributing to changes in CRC deaths attributable to a diet high in processed meat in China, stratified by sex. The bars illustrate the individual effects of aging, epidemiological change, and population growth on CRC deaths from 1990 to 2021. The black dots represent the combined effects of these three factors.

The decomposition analysis of CRC deaths attributable to a diet high in processed meat in China from 1990 to 2021 highlights the contributions of aging, epidemiological changes, and population growth to mortality trends (Figure 8). Aging was associated with a reduction in CRC deaths, while epidemiological changes and population growth contributed to an increase in deaths over this period. The combined effects of these factors, represented by black dots, show an overall rise in CRC mortality. All three factors (aging, epidemiological changes, and population growth) had a more pronounced effect on males than on females, indicating that men experienced greater changes in CRC mortality due to these contributing factors.

## Discussion

This study presents a detailed analysis of the burden of CRC attributable to processed meat consumption in China from 1990 to 2021, revealing significant findings. CRC mortality and the overall disease burden increased substantially during the study period, with age-standardized rates for deaths, DALYs, YLDs, and YLLs showing pronounced upward trends, particularly among males. The joinpoint analysis identified periods of rapid increases in CRC burden, most notably between 2007 and 2011. Age-period-cohort analysis demonstrated that mortality was higher among individuals diagnosed in recent periods and in more recent birth cohorts, suggesting that younger generations and those diagnosed more recently are at greater risk. This could be attributed to a growing prevalence of processed meat consumption in China, coupled with other lifestyle and environmental factors. The decomposition analysis further indicated that while population growth and epidemiological changes contributed significantly to the increase in CRC deaths, aging had a relatively smaller impact. These findings highlight an urgent need for public health interventions to reduce processed meat consumption and address the rising CRC burden in China.

The global burden of CRC attributable to processed meat consumption has been well established, but the situation in China is particularly concerning. Our study found that the CRC burden in China has increased substantially from 1990 to 2021, particularly among males. Age-standardized mortality and DALY rates for CRC attributable to processed meat consumption rose sharply after 1998, especially between 2007 and 2011. This trend differs from many Western countries, where CRC incidence and mortality related to processed meat consumption have stabilized or even decreased due to improved public awareness, dietary guidelines, and screening programs (13, 21). In China, rapid urbanization and economic development have led to increased consumption of processed foods, including processed meats, a shift that is reflected in the higher CRC burden (22). High processed meat intake was a main risk factor for CRC with a PAF of 8.6% (23). This suggests that public health efforts to reduce processed meat intake in China have not been as effective as in other countries, where dietary education and stricter regulations have mitigated the risks (21, 24).

The potential mechanisms underlying colorectal carcinogenesis due to processed meat consumption are well supported by biological and epidemiological evidence. Processed meat contains carcinogenic compounds such as nitrates, nitrites, HCAs, and PAHs, which are formed during processing and high-temperature cooking (1, 25). These compounds can cause DNA damage, promote inflammation, and accelerate tumorigenesis in the colon and rectum (1). Regular consumption of processed meat has been linked to an increased risk of CRC in multiple studies, making it one of the leading modifiable risk factors (26–28) and critical poor prognostic factors (29). Each daily 50-gram (approximately 1 serving) increase in processed meat intake was associated with an 18% increased risk of CRC (21). A 1% reduction in processed meat consumption might lead to 406 fewer CRC cases annually, while a 15% decrease could potentially reduce cases by 2,086 (30). Prevention efforts should focus on reducing processed meat consumption, particularly in rapidly urbanizing countries like China. Policies that encourage healthier diets, higher in fiber and lower in processed foods, and tighter regulation of processed meat products could play a critical role in reducing the CRC burden.

A significant finding in our study was the sex disparity in CRC burden, with males experiencing higher mortality and DALY rates compared to females. This aligns with previous studies showing that men tend to consume more processed meat and exhibit higher rates of CRC (31, 32). Biological differences also contribute to the sex disparity, with hormonal influences and differences in metabolism playing a role in CRC development (33). In addition, men might have higher exposure to risk factors associated with processed meat consumption due to lifestyle differences, such as eating habits, smoking, and alcohol consumption, which are known to interact with dietary carcinogens (34). Tailoring public health messages to address these sex differences, by encouraging dietary changes among men, could help reduce the CRC burden more effectively.

The results from our age-period-cohort analysis revealed that individuals diagnosed with CRC in more recent periods and those from more recent birth cohorts exhibited higher mortality rates. This indicates that CRC risk has increased across generations, with younger populations facing greater exposure to processed meat early in life. Recent cohorts have experienced faster dietary transitions, with processed meats becoming more prevalent in their diets, potentially leading to earlier and more frequent exposure to carcinogenic compounds. This cohort effect reflects the growing influence of Westernized diets in China, mirroring trends seen in other developing countries undergoing rapid dietary transitions (35). The period effect may also reflect broad societal changes, including greater access to processed foods and less engagement with traditional, plant-based diets. The rapid rise in CRC mortality among recent cohorts calls for urgent public health interventions focused on dietary changes, particularly among younger populations. The decomposition analysis highlighted that the increase in CRC deaths attributable to processed meat consumption was driven primarily by epidemiological changes and population growth, with aging contributing to a decline in deaths. While aging typically increases the risk of cancer, the reduction in CRC deaths due to aging could be attributed to improvements in early detection, treatment advances, and better management of other risk factors, such as smoking cessation and physical activity (36). However, the overall rise in CRC burden remains significant, driven largely by the expanding population and dietary shifts. Population-wide interventions aimed at reducing processed meat consumption and promoting healthier dietary patterns could substantially mitigate future increases in CRC mortality.

Given the rising burden of CRC attributable to processed meat consumption in China, effective public health interventions are critical to mitigating future disease risk. Regulatory measures should be prioritized, including the establishment of stricter guidelines on processed meat production and consumption. Policymakers could enforce limits on carcinogenic additives such as nitrates and nitrites while implementing mandatory labeling to inform consumers of the health risks. Economic policies, such as taxation on processed meat products, have been effective in reducing the consumption of harmful dietary components in other countries and could be considered as a strategy in China. Additionally, providing subsidies for healthier protein sources, such as poultry, fish, and plant-based proteins, may encourage dietary shifts away from processed meat. Beyond policy regulations, large-scale public health education campaigns are essential to raising awareness of the link between processed meat consumption and CRC. Integrating evidence-based dietary recommendations into national health guidelines, school curricula, and workplace wellness programs can promote informed decision-making. Given the observed gender and age disparities in CRC burden, targeted health education initiatives should be directed toward high-risk populations, particularly middle-aged and older men, who tend to consume processed meat more frequently. Future research should focus on evaluating the effectiveness of these interventions and monitoring dietary patterns to guide further policy refinements.

Despite providing valuable insights into the burden of CRC attributable to processed meat consumption in China, this study has several limitations. First, it relies on data from the GBD database, which integrates primary data and modeled estimates. While this approach ensures broad coverage, it may introduce uncertainties, particularly in regions with incomplete or heterogeneous reporting systems. Second, dietary exposure estimates in the GBD study are largely based on self-reported surveys, which are subject to recall bias and misclassification, potentially affecting the accuracy of processed meat intake assessment. Third, this study does not account for other lifestyle factors such as physical activity, smoking, and alcohol consumption, which are known to interact with diet and modulate CRC risk, potentially leading to residual confounding in the attributable burden estimates. Fourth, the analysis is conducted at the national level, which may obscure important subnational variations in CRC burden due to differences in dietary habits, healthcare accessibility, and socioeconomic factors. Future research should incorporate more objective dietary assessments, such as biomarkers, to improve exposure measurement accuracy, and adopt longitudinal designs to better capture the long-term effects of processed meat consumption on CRC risk while accounting for potential confounders. Additionally, regional analyses within China are warranted to identify high-risk populations and guide targeted public health interventions.

## Conclusion

This study underscores the importance of addressing the growing public health challenge posed by CRC linked to processed meat consumption in China. Public health strategies aimed at reducing processed meat intake, along with promoting healthier dietary patterns, are essential to mitigate the rising CRC burden. Future research should focus on improving the accuracy of dietary assessments and exploring regional variations within China to better understand the disparities in CRC risk. Additionally, more longitudinal studies are needed to evaluate the long-term effects of dietary habits on CRC development, accounting for other lifestyle factors. Public health interventions must be strengthened, with an emphasis on early education, policy implementation, and increased awareness of dietary risks. With a better understanding of modifiable risk factors and the impact of dietary transitions, future research can guide the development of targeted prevention strategies to reduce CRC incidence and mortality in China.

## Data Availability

Publicly available datasets were analyzed in this study. This data can be found at: http://ghdx.healthdata.org/gbd-results-tool.
